# *Ex-Vivo* Force Spectroscopy of Intestinal Mucosa Reveals the Mechanical Properties of Mucus Blankets

**DOI:** 10.1038/s41598-017-07552-7

**Published:** 2017-08-04

**Authors:** Javier Sotres, Skaidre Jankovskaja, Kristin Wannerberger, Thomas Arnebrant

**Affiliations:** 1 Biomedical Science, Faculty of Health and Society, Malmö University, 20506 Malmö, Sweden; 2 Biofilms-Research Center for Biointerfaces, Malmö University, 20506 Malmö, Sweden; 3 Ferring International Center SA, CH-1162 St-Prex, Switzerland

## Abstract

Mucus is the viscous gel that protects mucosal surfaces. It also plays a crucial role in several diseases as well as in mucosal drug delivery. Because of technical limitations, mucus properties have mainly been addressed by *in-vitro* studies. However, this approach can lead to artifacts as mucus collection can alter its structure. Here we show that by using an implemented atomic force microscope it is possible to measure the interactions between micro-particles and mucus blankets *ex-vivo* i.e., on fresh excised mucus-covered tissues. By applying this method to study the small intestine, we were able to quantify the stiffness and adhesiveness of its mucus blanket at different pH values. We also demonstrate the ability of mucus blankets to bind and attract particles hundreds of µm away from their surface, and to trap and bury them even if their size is as big as 15 µm.

## Introduction

Body cavities exposed to the external environment are lined by the so-called mucosal barriers i.e., one or more layers of mucus-covered epithelial cells overlying loose connective tissue. These barriers act as the primary interface between the host and the physical environment. Mucus i.e., the viscous and hydrated gel that covers epithelial surfaces serves multiple purposes^1, 2^. It is a lubricious barrier that protects the epithelium from harsh chemical conditions, vigorous shearing actions and pathogenic organisms. Nevertheless, mucus still allows, in an extremely selective manner, the diffusion of e.g., ions and nutrients. Mucus also keeps the underlying epithelium hydrated and serves as a niche for commensal bacteria. Historically, most of the research on mucosal barriers has focused on the epithelial cells and on the underlying lymphoid tissues. In comparison, mucus has traditionally received much less attention. However, this tendency is changing. It is now well-established that many mucosa diseases e.g., cystic fibrosis, ulcerative colitis and even the propensity to certain infections, are related to dysfunctions of the mucus barrier such as mucus hypo or hypersecretion and defects of its main solid component i.e., mucins^3–5^. Whether these dysfunctions are consequences or causes of the diseases, further research in this direction will increase our understanding on the corresponding etiology and could lead to novel therapeutic strategies. Mucus also plays a crucial role in mucosal drug delivery as it is the first barrier faced by drugs or drug delivery systems prior to absorption. While this aspect has been as well traditionally underestimated, the need for a deeper mechanistic understanding of mucus as a barrier to drug delivery is nowadays well-recognized^6, 7^.

The difficulty of performing *in-vivo* and *ex-vivo* mucus studies is probably behind the fact that mucus has been less investigated than the underlying mucosal layers. Nevertheless, *in-vitro* studies are numerous, and many indicate that the physico-chemical properties of mucus are of direct relevance to its physiological functions^8^. In most cases, mucus can be pictured as a thixotropic aqueous gel composed primarily of crosslinked and entangled mucins i.e., the main solid component of mucus^9^. These are long glycoproteins with a central region that contains a high density of oligosaccharide side-chains (accounting for ca. 80% of their mass) that, by steric interactions, stretch away in a bottlebrush configuration^10^. Their hydrophobic and low glycosylated terminal regions are responsible for mucin self-association and, subsequently, for the formation of the mucus matrix^10^. The rheology of the resulting gel has been extensively studied^9^. At the macro-scale mucus exhibits shear-thinning properties. Mucus macro-rheological properties also determine the diffusion of particles bigger than the mesh spacing of the mucin network, for which typically a value close to 1 µm is reported^1^. For non-mucoadhesive particles smaller than the mucus mesh spacing mucus exhibits a similar viscosity as water^9^. Not unexpectedly, the physico-chemical properties of mucus are drastically dependent on the ambient medium. For instance, lowering the ambient pH leads to mucin aggregation and, therefore, to an increase of mucus viscosity^11, 12^. This mechanism might be of physiological and clinical relevance as drops in luminal pH might occur as a consequence of different diseases, physiological states and bacterial fermentation of carbohydrates^13^.

*In-vitro* studies i.e., performed on collected and even purified mucus, have provided valuable knowledge. However, it cannot be expected that they give the true picture of the *in-vivo* situation. Mucus collection methods exert mechanical stimulation that can induce considerably alteration of its structure^9^. Moreover, purification protocols usually lead to mucin degradation^14^. These problems can be avoided by studying (*ex-vivo*) freshly excised mucosal samples. However, this approach also has difficulties, most often due to the incompatibility of experimental techniques and the softness of mucosal tissues. Indeed, *ex-vivo* physico-chemical studies are scarce^15^ and most often focus on mucoadhesion at the macro-scale^16^. Thus, there is a high need for a tool for *ex-vivo* physico-chemical studies of mucus blankets.

In the study of the mechanical properties of mucus, the Atomic Force Microscope (AFM) deserves a special mention. AFM is not only a microscope with sub-nm resolution. It can also be used to investigate forces between micro- or nano-meter sized probes and surfaces with a pN resolution. This technique, known as force spectroscopy, has been extensively used to study the mechanical properties of mucin films^16–18^. However, AFM has suffered from the same drawback stated above: it has not been possible to use it to study mucus-covered tissues. AFM setups use piezo-actuators to control with high precision the relative positioning between probe and sample. Most piezo-actuators allow vertical displacements of a maximum of a few µm. Even though some available implementations increase this range up to distances of ca. 100 µm^19^, this is still below the distance range for which a probe and a mucus covered tissue interact as shown in this work.

Here, we show how a standard AFM setup can be modified to overcome this technical limitation allowing *ex-vivo* studies of mucosal tissues. The technique was applied to study the interactions between micro-particles and the mucosal surface of the distal part of the small intestine (ileum) collected from adult pigs. The choice of pig as the source of ileum samples relied on the fact that it is a human-sized omnivorous animal which possesses similar digestive and associated metabolic processes as humans. Moreover, mucus from pig and human sources exhibit similar viscoelasticity^9^. By focusing on ileum we made sure that our investigations were not influenced by the structural differences of mucosa with respect to its location. Moreover, the barrier properties of ileum mucosa are critical in many aspects. Ileum hosts most of the bacterial population of the small intestine^20^. Alterations in the barrier function of ileum could also be in the origin of inflammatory bowel diseases such as Crohn’s disease^5^. Additionally, the presence of Peyer’s patches in the ileum makes this section a strategic target for orally administrated drugs^21^. The understanding and control of these aspects will definitely benefit from the development of experimental methods for the study of the physico-chemical properties of this biological interface. Here we show that AFM-based force spectroscopy can be used for this purpose. Specifically, we were able to quantify, at different pH values, the stiffness and adhesiveness of ileum mucosa. Additionally, we showed that the outer mucus blankets can give rise to bridging interactions that extend several hundreds of µm from their outer steric repulsive surface, and how this outer surface can absorb particles as big as 15 µm i.e., more than one order of magnitude bigger than the values typically reported for mucus mesh size^1^.

## Results

### Scanning Electron Microscopy of Ileum Mucosal Surfaces

**Figure 1:**
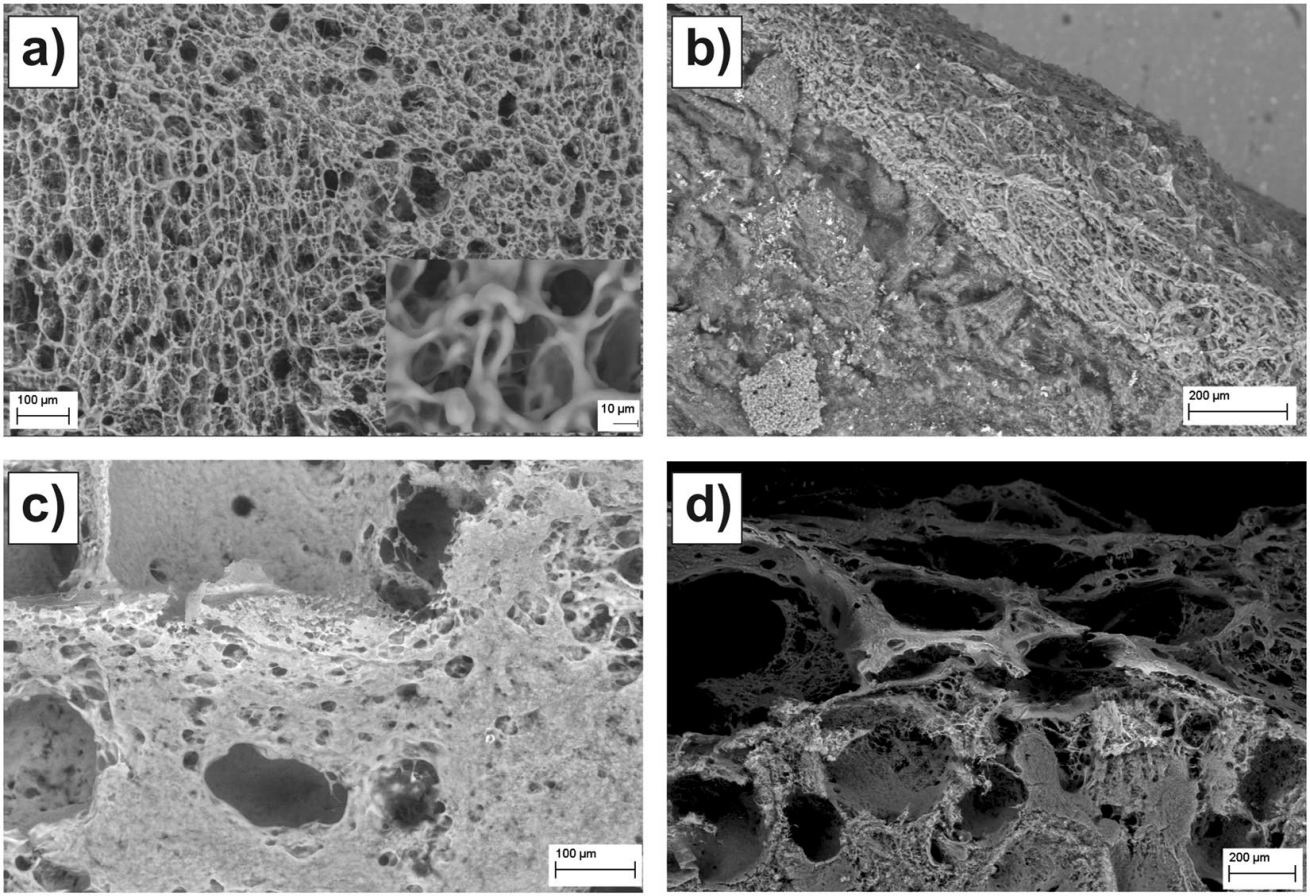
SEM images of ileum mucosa. (**a)** Top and (**b)** section images of ileum samples exposed to PBS pH 7.4 for 30 min. The inset in (**a**) shows the same sample at a higher magnification. (**c)** Top and (**d)** section images of ileum samples subsequently exposed to PBS pH 2 for 30 min.

SEM was used to visualize vitrified and freeze-dried ileum samples. Samples which were previously exposed to PBS buffer pH 7.4 for 30 min (Fig. 1a and b) exhibited a network structure on their outermost surface (Fig. 1a) which homogeneously covered the intestinal villi forming the epithelium surface (Fig. 1b). This network exhibited an average outer mesh size in the 10–30 µm range. However, this value significantly decreased along with depth (inset, Fig. 1a). Significant structural changes were observed if, after the exposure to the physiological solution, these samples were subsequently exposed to an acidic environment (PBS solution pH 2 for 30 min). In this case, SEM visualization revealed significantly more compact mucus blankets (Fig. 1c and d).

### Force Spectroscopy of Intestinal Mucosal Surfaces

**Figure 2:**
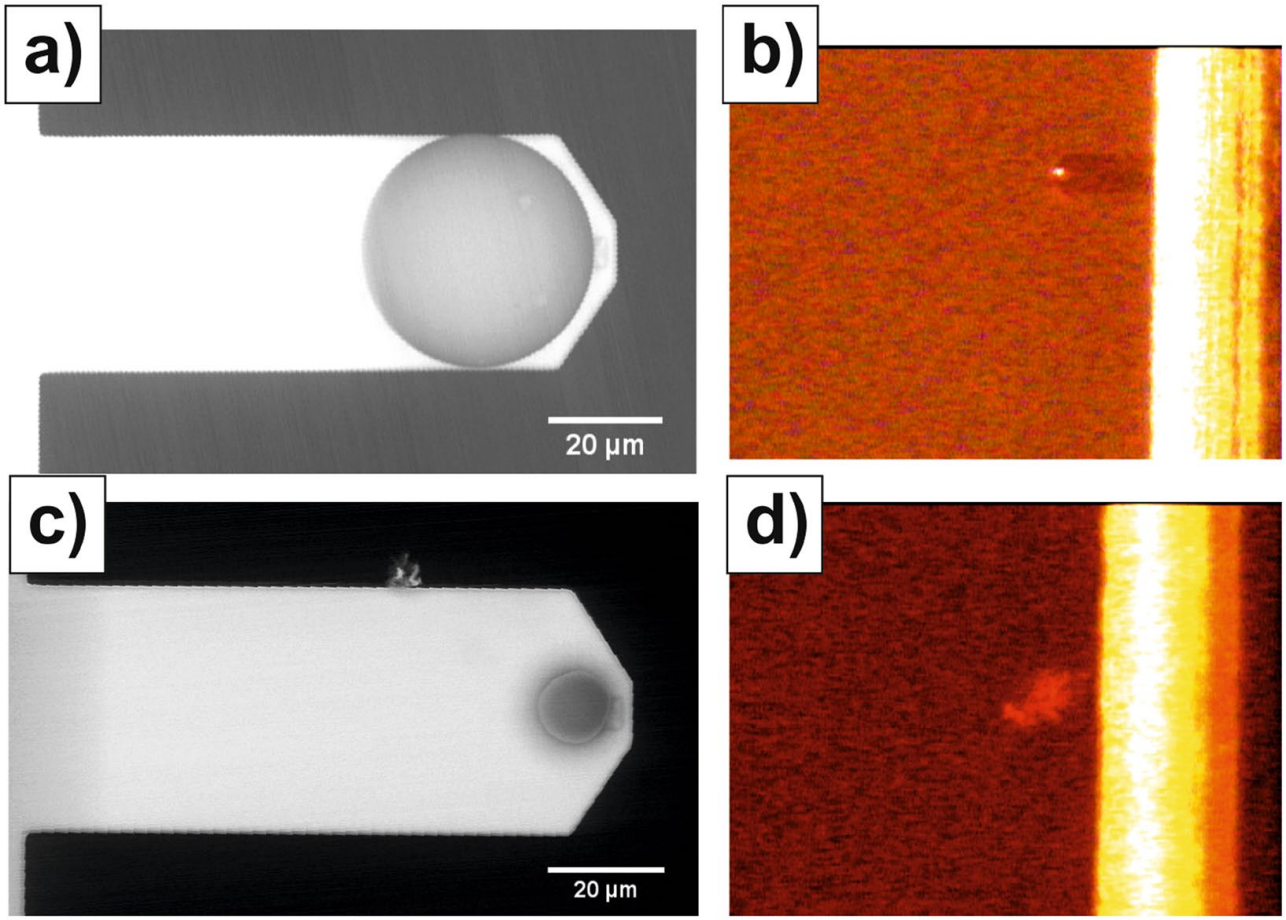
Role of probe size in the interaction with mucus blankets. **(a**) Representative SEM image of the 40 µm colloidal probes used in this study (the actual diameters were always determined from the analysis of the SEM images). (**b**) Optical image registered when the probe shown in (**a**) established contact with ileum mucosa at a repulsive force of 10 nN. **(c)** Representative SEM image of the 15 µm colloidal probes used in the study. (**d)** Optical image registered when, while approaching ileum mucosa with the probe shown in (**c**), the signal registered by the PSD dropped to zero.

The interaction between fresh ileum mucosal surfaces and silica colloidal probes was investigated by means of AFM-based force spectroscopy. The size of the probes was revealed as a critical parameter in these experiments. Specifically, we investigated these interactions using cantilevers where probes of nominal diameter 40 µm and 15 µm were attached to their free ends (Fig. 2a and c respectively, the actual diameters were always determined by SEM imaging). When approaching the samples with the colloidal probes, the contact with the intestinal surface should be reflected by an increase in the vertical deflection of the cantilever as registered by the AFM Position Sensitive Detector (PSD). Indeed, this was the case when 40 µm probes were employed. However, when approaching the samples with the with 15 µm probes, the total intensity registered by PSD dropped to zero before a significant change in the cantilever deflection could be observed. Optical monitoring of the cantilever during the experiments provided further insight. For the 15 µm probes, the drastic drop in the PSD signal was the result of the laser beam being scattered (Fig. 2d). This indicates that the probe penetrated the mucus blanket which ended covering and burying even the whole cantilever and, therefore, scattering the laser beam. Indeed, after retraction from the samples, cantilever contamination was evident from the optical images. A similar scattering was not observed for the 40 µm probes indicating that they did not penetrate the mucus blankets (Fig. 2b), at least for the maximum normal forces applied in this study (ca. 10 nN), but compressed them instead. Thus, they were used for further characterization experiments.

**Figure 3:**
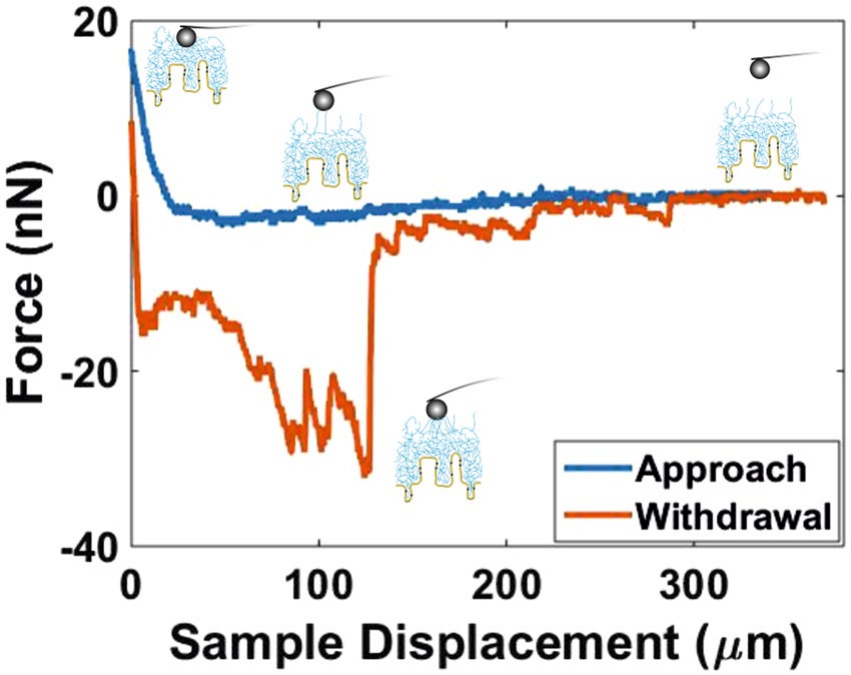
Representative force measurement on ileum mucosa in PBS pH 7.4.

A representative force vs. sample displacement measurement i.e., plot of the interaction force for consecutive approach and withdrawal displacements, obtained on ileum mucosa in PBS pH 7.4 is shown in Fig. 3. Two features of the force during approach are worth to mention. First, the presence of a long range attractive force which is felt by the probe hundreds of µm far from the repulsive contact with the mucosal surface. Electrostatic forces at physiological ionic strengths extend only up to few nm, so that an electrostatic origin can be discarded. Instead, this force can be ascribed to a bridging interaction. Secondly, the repulsive (contact) region of the approach force measurement revealed the significant softness of the samples, which could be compressed by tens of μm for applied forces in the order of 10 nN. The force measured while withdrawing probe and sample also revealed worth to mention features. First, a significant adhesion between probe and sample was observed. Secondly, hysteresis was observed in the contact region between the approach and withdrawal measurements. Irrespectively of the fact that both movements were performed at different speeds (see Methods section), this indicates that plastic deformation took place on the sample^22^. In order to avoid the influence on our data of the irreversible changes in mechanical properties that can arise from plastic deformations, each force measurement was performed on a different lateral position.

**Figure 4:**
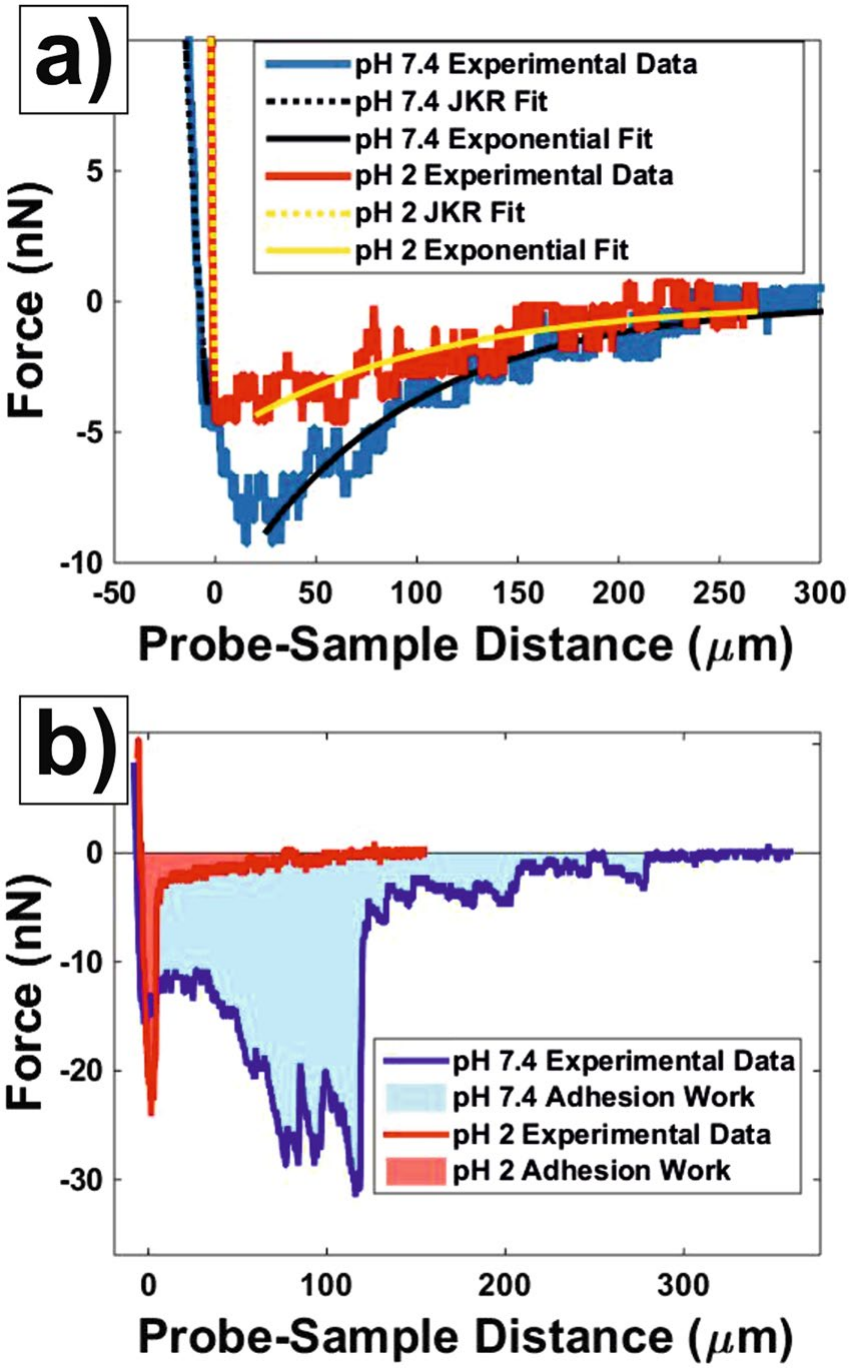
Representative force measurements on ileum mucosa at different pH values. (**a)** Approach force measurements obtained in PBS pH 7.4 (blue) and pH 2 (red). Fits of the contact region to the JKR model (Eq. 3) and of the non-contact region to an exponential function (Eq. 8) are also shown. (**b)** Withdrawal force measurements obtained in PBS pH 7.4 (blue) and pH 2 (red). The shaded area below the adhesion peaks corresponds to the work of adhesion.

**Figure 5:**
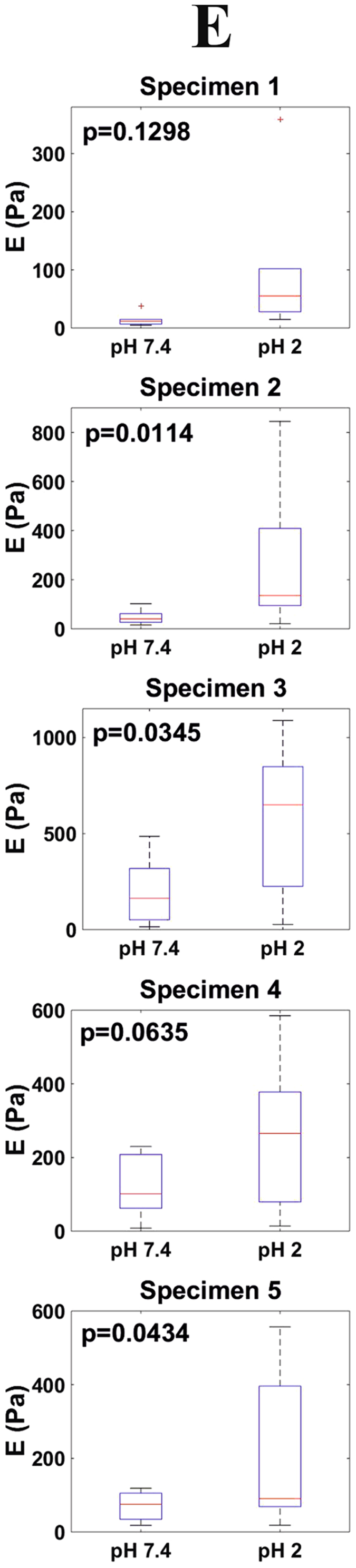
Distributions (n = 10) of E values obtained at both pH 7.4 and pH 2 for all investigated specimens.

We now focus on each of the mentioned interactions and on how they are affected by the ambient pH. Figure 4a shows approach force curves (in the force vs. probe-sample distance representation) obtained on ileum mucosa both in PBS pH 7.4 and PBS pH 2. The repulsive (contact) region of the curves was used to quantify the stiffness of the samples in terms of their Young modulus, E. For this, the JKR model^23^ (Eq. 3), which is considered valid for compliant samples, large tip radii, and high adhesion forces, was used. Fits to the JKR model are also shown in Fig. 4a for the two plotted force measurements. The corresponding E distributions obtained for all (a total of five in this work) investigated specimens at both pH values are shown in Fig. 5. E median values at pH 7.4 laid within the range 11–163 Pa for all the studied specimens. In all cases, lowering the ambient pH to 2 led to an increase in the E median values by a factor between 1.2 and 4.7, a difference that was significant (p < 0.1) for four out of five specimens.

**Figure 6:**
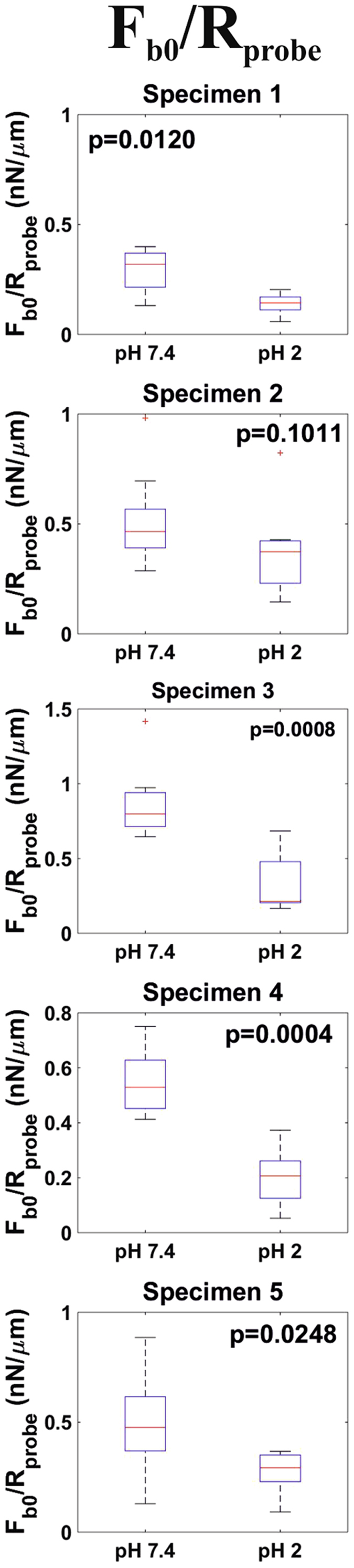
Distributions (n = 10) of Fb0/Rprobe values obtained at both pH 7.4 and pH 2 for all investigated specimens.

**Figure 7:**
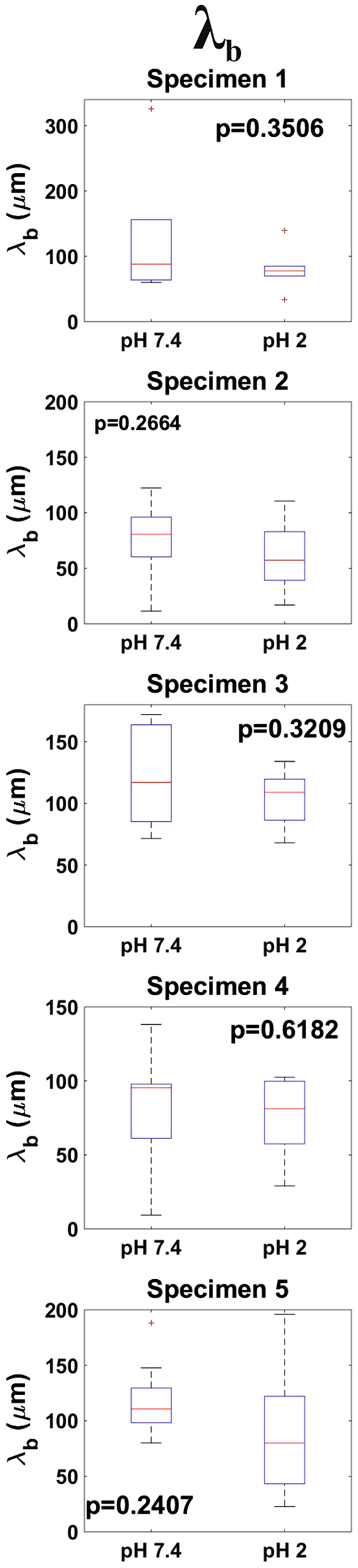
Distributions (n = 10) of λb values obtained at both pH 7.4 and pH 2 for all investigated specimens.

Following previous studies^24, 25^, the long-range attractive bridging forces measured while approaching probe and sample were fitted to a single exponential function (Eq. 8). From this fit, both the amplitude, F_b0_, and the characteristic length, λ_b_, of the force were obtained. In Fig. 4a it can be seen how this model provided a good fit to the non-contact region of approach force curves measured both at pH 7.4 and pH 2. In order to account for the effect of the probe size, the strength of the bridging interaction was characterized by F_b0_ escalated by the radius of the probe R_probe_, The distributions for F_b0_/R_probe_ and λ_b_ are shown in Figs 6 and 7 respectively. F_b0_/R_probe_ median values at pH 7.4 laid within the range 0.32–0.80 nN/µm. For all specimens, lowering the pH to 2 led to a decrease of F_b0_/R_probe_ by a factor in the 1.2–3.8 range. This difference was significant (p < 0.1) for four out of five specimens. Regarding λ_b_, at pH 7.4 median values in the 80–111 µm values were found. This quantity decreased for all specimens by lengths in the 8–31 µm range when probed at pH 2. However, this difference could not be proved to be significant for any of the investigated specimens. However, it cannot be discarded that this was just an artifact resulting from the small number of measurements (n = 10) that could be performed on each sample.

Ileum samples extensively washed with deionized water were also investigated (Supplementary Information, Section 1). Whereas, as shown in Fig. 1, the mucus blankets of ileum samples were preserved when gently washed for short time periods with solutions of physiological ionic strength, this was not the case when extensively rinsed with deionized water. Indeed, this procedure completely removed the mucus blankets. When investigated by means of force spectroscopy, samples lacking the mucus blankets revealed a significantly higher stiffness (E in the 14–22 kPa range) and an absence of long-range bridging interactions. This indicates that, when present, mucus blankets are the main responsible for the softness and bridging interactions exhibited by mucosal surfaces.

**Figure 8:**
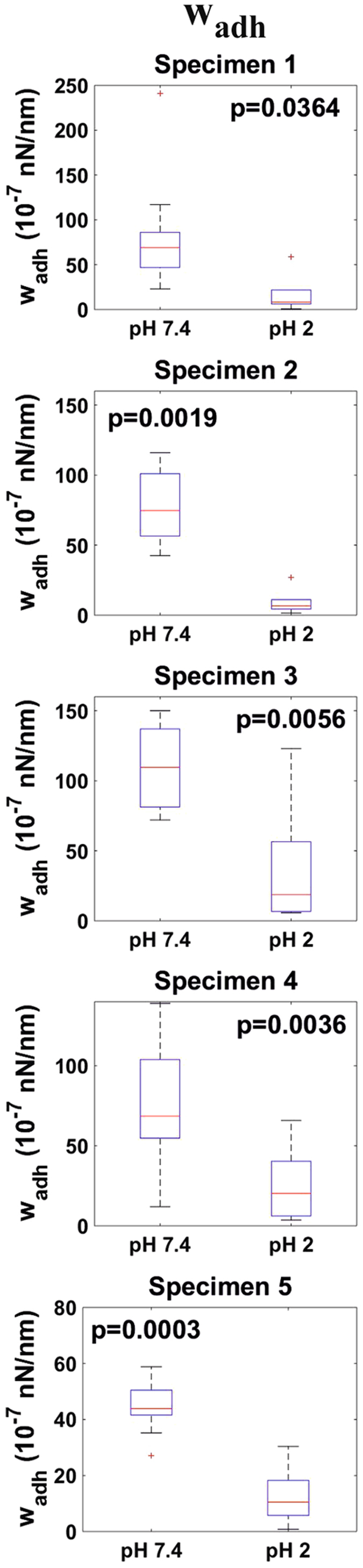
Distributions (n = 10) of wadh values obtained at both pH 7.4 and pH 2 for all investigated specimens.

Representative withdrawal force measurements (in the force vs. probe-sample distance) obtained on ileum mucosa at pHs 7.4 and 2 are shown in Fig. 4b. These curves show a significant adhesion. This adhesion was quantified in terms of the adhesion energy per unit area, w_adh_ (Eq. 10). The distributions of w_adh_ obtained for both studied pHs on all specimens are shown in Fig. 8. w_adh_ median values at pH 7.4 laid within the range 69–110 10^−7^nN/nm for all the studied specimens. In all cases, lowering the ambient pH to 2 led to a decrease of w_adh_ median values by a factor between 3.4 and 12.8. A two sample t-test revealed that this difference was very significant (p-value < 0.05) for all specimens.

Of relevance for the analysis of the results is that exposure of ileum samples to physiological buffer for several hours eventually led to the detachment of their mucus blankets (Supplementary Information, Section 2). It is reasonable to assume that this is a gradual process and, therefore, its influence on our data needs to be considered. However, this was not the case. The reported experiments were performed within 5.5 h of the sacrifice of the specimens and within 2.5 h of the removal of the luminal content. The data obtained within this timeframe did not exhibit a significant correlation with acquisition time (Supplementary Information, Section 3). Thus, we can conclude that ageing-induced mucus-detachment did not significantly influence our results.

## Discussion

AFM has been extensively used in the study of biological material such as lipids, proteins, viruses, bacteria and eukaryotic cells^26^. However, there are still only few works where it has been applied to study biological tissues^27, 28^, and so far none where the sample of study was mucus-covered tissues. Probably, the main limiting factor has been the high softness and penetrability shown in this work for these samples. When pressed by AFM probes they deform by distances larger than those achievable by common AFM positioning systems, often piezo-actuators. The aim of using piezo-actuators is to achieve a sub-nm resolution in the probe-sample separation. However, this is not strictly needed when the interactions take place over distances in the order of hundreds of µm, like in the case where mucus-covered tissues are probed. As showed, in this situation the probe-sample separation can be controlled with a motor driven micrometer screw with resolution enough for the characterization of the forces developed at the nN level. Additionally, by using sufficiently big probes, in our work ca. 40 µm, the penetration exhibited by smaller probes could be avoided and the mucosal surfaces could be compressed instead. In this way, we showed that AFM-based force spectroscopy can be used for *ex-vivo* studies of the mechanical aspects of mucus-covered tissues. Specifically, it could be used to study their stiffness, in terms of the Young modulus, their adhesiveness, in terms of their adhesion energy, and even their outer structure, as inferred from the presence of bridging interactions and the penetration of the 15 µm probes.

The presented methodology opens completely new possibilities in mucus research. However, in its current state it lacks the ability to obtain a statistically significant number of measurements i.e., a number that would result in smooth distributions of the quantities derived from the force measurements, before mucus blankets undergo ageing-induced structural changes. As detailed in the Methods section, this is a consequence of the acquisition process being time-consuming. At present we are working on a computer-positioning system interface that will reduce the acquisition time and, therefore, improve statistics. Additionally, this interface will allow controlling the approach and separation velocities as well as the contact time. This will allow viscoelasticity studies as well as a more thorough characterization of mucoadhesion processes^29, 30^.

The JKR model provided a good fit to the contact region of force measurements on ileum mucosa. This allowed the estimation of its Young modulus, E. One of the main uncertainties of stiffness measurements on multi-layered samples is to determine how each of the layers contribute to the measured value. This is the case of the presented experiments as the gastrointestinal wall is made by several tissue layers, the outermost one i.e., mucosa, being covered by a mucus blanket. In this work, the Young modulus values obtained for all specimens and pH values (Fig. 5) were within the range 11–266 Pa. When it comes to biological materials, these values are significantly low e.g., when compared to those reported from AFM experiments on cells which are typically of the order of kPa^31^. Moreover, force measurements on mucosa ileum samples where the mucus blankets were completely removed resulted in Young moduli in the 14–22 kPa range (Supplementary Information, Section 1). This indicates that in our experiments the main contribution to the measured stiffness came from the mucus blankets which completely covered the samples (Fig. 1). From a practical aspect, the fact that the presence of mucus blankets led to a decrease between two and three orders of magnitude of the effective Young modulus of the ileum indicates that the energy resulting from mechanical insults will be primarily absorbed by the mucus blankets, protecting in this way the underlying fragile epithelium.

We also showed that the presented methodology can be used to measure *ex-vivo* the adhesive interactions between micro-particles and the ileum mucosa. The adhesion events extended up to several hundreds of µm for the repulsive steric surface and exhibited a non-linear shape which is representative of polymer extension profiles^32^ (we were even able to identify several adhesion peaks, Fig. 4b, that could be attributed to the dissociation of a few, if not single bonds). This, along with the fact that the outer surface consisted of mucus blankets (Fig. 1), indicates that the rupture events are preceded by the extension of mucin complexes. Our results open the possibility to study at the single particle level the interactions between mucus blankets and mucoadhesive particles, which is considered one of the most promising strategies for controlled drug release in the gastrointestinal tract^33^. We anticipate that this will provide insight into the underlying mechanisms which are hindered when using macroscopic mucoadhesion techniques.

This work shows that force spectroscopy is sensitive to modifications in the mechanical properties of mucus blankets induced by changes in their environment, specifically in the ambient pH. How the mucus blankets are influenced by pH is of relevance in many situations. The most obvious one is the exposure of gastric mucus to hydrochloric acid in the stomach during digestion^34^. The esophagus can also be exposed to an acidic environment as a consequence of reflux events^35^. Localized decreases of intestinal pH have also been reported as a consequence of different diseases^13^ and of bacterial fermentation of carbohydrates^36^. As a proof of concept, we investigated the changes in mucus when lowering the pH from a physiological value (7.4) to an acidic value (2) where the groups mostly responsible for the charge of mucins i.e., sialic acids (pKa≈2.6), would be neutralized. Lowering the ambient pH from 7.4 to 2 had a clear impact on mucus structure as revealed by SEM imaging (Fig. 1). Whereas it is difficult to determine the influence of freeze-drying on the samples, is at least clear that acidic pH led to compacter mucus blankets. This is supported by force spectroscopy data. Decreasing the ambient pH from 7.4 to 2 led to stiffer samples i.e., their Young modulus increased by a factor between 1.2 and 4.7. Thus, when exposed to an acidic solution, mucus blankets become compacter and harder indicating a mechanism by which they could protect the underlying epithelium from such a harsh environment. Interestingly, SEM imaging also revealed a higher lateral in-homogeneity for the structure of mucus exposed to an acidic environment (Fig. 1). Force spectroscopy data suggests that this is not only an artifact of the sample preparation procedure required for SEM imaging. Indeed, the distribution of measured Young modulus values were significantly broader when probed under acidic conditions (Fig. 5). As force measurements were always obtained on different lateral positions, this supports that exposure to an acidic environment leads to more laterally inhomogeneous mucus blankets.

Decreasing the pH did not only lead to stiffer but to less adhesive mucus blankets as well i.e., the adhesion energy per unit area decreased by a factor between 3.4 and 12.8 when lowering the pH from 7.4 to 2. Both results agree with mechanisms reported from *in-vitro* experiments on mucin gels, where a decrease in pH led to an increase in viscosity^11, 12, 37^ and aggregation^38^. Mucin films at solid-liquid interfaces also become more compact at low pH values^18^. This transition is driven by i) the reduction of electrostatic repulsion between the anionic moieties of the mucin molecules (mostly sialic acids) which in turn ii) allows the interaction and subsequent aggregation of their hydrophobic residues. This scheme explains the higher stiffness that we observed at low pH. It also explains the lower adhesiveness. Most likely the silica micro-particles did not adhere to the oligosaccharide chains of the mucins because both being highly hydrophilic and of similar isoelectric point. Thus, adhesion was probably a consequence of the binding of the micro-particles to the hydrophobic mucin residues, or other mucus components coupled to them. Under acidic environment, these residues would aggregate so that less of them would be available to interact with the micro-particles. Thus, our results support the validity of mucin gels as model systems for the study of the physico-chemical properties of mucus. This result is not to be underestimated as it has been a matter of controversy^39, 40^. The fact that the method presented here has a minimal invasiveness, suggest that other contradicting results might be influenced by mucus collection procedures which might alter mucus original structure and composition (e.g., by collecting mucosal cellular material as well).

Beside the measurement of mucus stiffness and adhesiveness, we present two aspects of mucus blankets not observed so far: (i) the presence of long range (ca. 100–300 µm) bridging interactions and (ii) the unopposed penetration of mucus blankets by 15 µm particles. The occurrence of bridging interactions indicates the presence of a layer consisting of a very low density of long and flexible mucin complexes that stochastically extend out of their equilibrium conformation and that can, therefore, interact with features hundreds of microns far from the mucus outer steric repulsive surface. The attractive character of these interactions indicate that the mucin complexes could bind the silica probes. Eventually, the extended mucin complexes tend to recover their equilibrium conformation i.e., the resulting gain in entropy dominates over steric repulsion, resulting in the attractive nature of the interaction. This is in accordance with the adhesion observed in withdrawal force measurements. Following previous studies^24^, bridging interactions were fitted with an exponential function. This provided both the characteristic length, λ_b_, and the amplitude (strength) of the interaction, which was scaled by the radius of the probe in order to account for its size, F_b0_/R_probe_. Thus, F_b0_/R_probe_ provides an estimation of the number of mucin complexes that could bind the probes and on the strength of this binding. It is of relevance that F_b0_/R_probe_ data is in accordance with adhesion measurements i.e., both F_b0_/R_probe_ and w_adh_ exhibited a significant decrease when the ambient pH was lowered from 7.4 to 2. The median values obtained for λ_b_ also decreased with pH, even though this decrease could not be proved to be statistically significant. Nevertheless, this is probably just a consequence of the low number of measurements as a decrease in λ_b_ would be in agreement with the acidic pH-induced compaction of mucus blankets discussed above. It has been reported that when in good solvent conditions polymer bridging interactions exhibit an exponential behavior, the characteristic length of the interaction is close to the radius of gyration, R_g_, of the polymers^41^. This suggest a R_g_ value for the mucin complexes that form ileum mucus blankets in the order of ca. 100 µm. Once the bridged complexes reach their equilibrium conformation, a purely repulsive steric contact is expected. Indeed, this was observed for the 40 µm probes. However, the 15 µm probes were completely immersed in the mucus before a significant repulsive interaction could be registered. This indicates that the outer mucus mesh size lies in the 15–40 µm range. This range of values is significantly bigger than those reported in the literature i.e., 0.5–1 µm^1^. Probably, the discrepancy arises from the fact that mesh sizes are typically measured for the bulk mucus by means of micro-rheological investigations. Instead, in our experiments we probed the very outer section of mucus blankets. Thus, this indicates that mucus blankets are characterized by a gradient in their mesh size. This is also supported by SEM imaging (Fig. 1a, inset). This observation, along with that of bridging interactions, picture a mechanisms by which intestinal mucus blankets could probe luminal content. By means of bridging interactions, mucin complexes can “fish” features even hundreds of microns away from the mucus outer surface. Subsequently, mucus blankets would be able to attract, trap and bury these features in their outer section even if their size is 15 µm (and probably even higher). Of relevance for the intestinal environment is that this size is higher than the values typically reported for most bacteria (ca. 1–10 µm). Mucus blankets could use this mechanism to e.g., sequester pathogenic bacteria from the lumen and subsequently expel them as mucus is transported distally by peristalsis.

## Methods

### Mucosa Samples

Fresh sections of the small intestine from adult pigs (ca. 6 months old) were acquired from a local abattoir immediately after the sacrifice of the specimens. No ethical approval was required as the specimens were sacrificed for a different purpose than those reported in this study. Immediately after the sacrifice, sections of the distal part of the small intestine (ileum) were removed by sharp dissection, opened longitudinally along the side opposite to the mesenteric attachment remnant and gently washed with PBS buffer pH 7.4 (P4417, Sigma-Aldrich, St. Louis, MO, USA) in order to remove luminal content. Then circular samples of an appropriate size for AFM and SEM experiments (ca. 1.6 cm diameter) were excised taking care of not removing the seromusculature layers. The time passed since the sacrifice of the specimens and the final step of sample preparation was kept below 3 h.

For AFM experiments, fresh excised sample were immediately investigated in PBS pH 7.4 for a maximum of 1 h. Afterwards, the samples were rinsed with, and kept in, PBS pH 2 (adjusted with HCl 0.12 M) for 30 min and subsequently investigated in this same solution for a maximum of 1 h.

### Scanning Electron Microscopy

An Environmental Scanning Electron Microscope (SEM) (EVO LS10, Zeiss, Germany) was used to visualize both colloidal probes and mucosa samples. For this, the SEM was operated in the variable pressure mode (10 Pa, EHT voltage 25 kV) and the images acquired using the backscattered detector.

For SEM experiments, fresh excised samples (less than 3 h since the sacrifice of the specimens) were subjected to three different treatments: (1) incubation in PBS pH 7.4 for 30 min, (2) incubation in PBS pH 7.4 for 30 min followed by incubation in PBS pH 2 (adjusted with HCl 0.12 M) for 30 min and (3) incubation in PBS pH 7.4 for 4 h. Immediately after, the samples were vitrified with liquid nitrogen and freeze-dried using a commercial setup (Alpha 1–4 LSC, Martin Christ Gefriertrocknungsanlagen GmbH, Germany). This consisted in a main drying step (1.030 bar, −25 C, 4 h) and a final drying step (0.001 bar, 20 C, 1 h). A razorblade was used to obtain sections of the dried samples. Dried samples were subsequently used for SEM visualization without further treatment.

### Colloidal probes

Rectangular silicon nitride cantilevers with a nominal normal spring constant of 0.76 N·m^−1^ were employed (OMCL-RC800PSA, Olympus, Japan). This spring constant might seem high for investigating the extremely soft intestinal mucosa. However, the use of softer cantilevers was not a viable option as their downward deflection while withdrawing (resulting from probe-sample adhesion) was higher than that which could be monitored with the AFM position sensitive detector (PSD). Spherical silica particles with a nominal diameter of 15 μm (PSI-15.0, G. Kisker GbR, Germany) and 40 μm (Corpuscular Inc., Cold Spring, NY) were attached to the free end of the cantilevers by using a two part epoxy adhesive (Loctite, Henkel Norden AB, Sweden) and by manipulating the cantilevers with the AFM with the help of an optical microscope (Nikon, Amsterdam, The Netherlands). Colloidal probes were visualized by variable pressure SEM for characterizing their shape and size.

### Force Spectroscopy

A commercial AFM setup equipped with a liquid cell (MultiMode 8 SPM with a NanoScope V control unit, Bruker AXS, Santa Barbara CA) was employed for the acquisition of force measurements. Force measurements were performed first by approaching and then by separating the mucosal surfaces and the AFM cantilever with the colloidal probe attached at its free end. These experiments required controlling the distance between probe and sample during much longer ranges than those attainable with commercially available AFM scanners. The separation between probe and sample was controlled by means of a motor-controlled leadscrew to lift the AFM head (i.e., the cantilever). Approach displacements were accomplished by controlling the leadscrew with the Engage option of the AFM software (NanoScope v8.10, Bruker AXS), which lifts the head downwards at a speed of ca. 5.14 μm/s. Withdrawal displacements were achieved by controlling manually the Motor Control Switch in the AFM head. This rotates the leadscrew, lifting the AFM upwards at a speed of ca. 70.90 μm/s. In this way it was possible to perform force measurements on mucosa samples by approaching and withdrawing the AFM probe by distances of several hundred μm. In AFM, cantilever deflection is usually monitored by focusing a laser on the top of its free end and registering the reflected beam with a position sensitive detector (PSD), specifically a four quadrant photodiode. In our case, the vertical and lateral signals on the PSD were monitored by means of a digital oscilloscope (TDS 2022C, Tektronix, Beaverton, OR). The contact time i.e., that between approach and withdrawal was set to 30 s and the applied normal force during this period was set to ca. 10 nN. It was not possible to study lower contact times as this was limited by the time needed to store the PSD signals in the oscilloscope internal memory after each vertical ramp.

In order to avoid ageing effects, only fresh samples (less than 3 h after the sacrifice of the specimens) were investigated, initially in physiological solution (PBS pH 7.4). Then, samples were exposed to an acidified PBS solution (pH2) and subsequently investigated in this same solution for a maximum of 1 h. Experiments could not be extended for a much longer time as the mucus blankets could eventually dissolve (Supplementary Information, Section 1). This experimental time constraint, along with the time required to perform a measurement (few minutes between completion of successive force measurements on different lateral positions), allowed the acquisition on average of only 10 force measurements on each sample on each of the tested solutions. Statistics could not be improved by investigating several samples from the same specimen as the time since the sacrifice of the animal and, therefore, the unavoidable ageing would have been different. Instead, results obtained on samples from different specimens were compared.

### Analysis of Force Spectroscopy Data

A self-written routine in Matlab (The MathWorks, Natick, MA) was used to analyze raw force spectroscopy data, which in our case consisted in vertical PSD signals monitored over time. First, time was converted into distance by scaling with the appropriate speed value (see above). Then, the PSD vertical signal corresponding to zero deflection was calculated by averaging its value over a window of positions far enough from the contact or adhesion regions, and subsequently subtracting this offset from the corresponding PSD vertical signal. The PSD vertical signal was then converted into cantilever vertical deflection units by scaling with a factor obtained from a linear fit of the contact region of force curves obtained on clean mica surfaces. The cantilever deflection, d, was related to the probed force, F, by the cantilever spring constant, k, obtained from thermal noise analysis^42^:

1 $$F=kd$$

For approach curves, the point where mechanical contact between tip and sample was established was found by fitting the contact region of the curve with the JKR elasticity model^23^. Specifically, the deformation of the sample, δ, can be expressed by:

2 $$\delta =z-{z}_{0}-d$$

where z is the sample displacement and z_0_ is the contact point. In the JKR model, the indentation for a sphere-planar geometry is given by:

3 $$\delta =\frac{{r}_{c}^{2}}{R}-\frac{4}{3}\sqrt{\frac{{r}_{c}{F}_{adh}}{RK}}$$

where R is the radius of the sphere, F_adh_ the adhesive force, and r_c_ is the contact radius which can be expressed as:

4 $${r}_{c}={[\tfrac{R}{K}{(\sqrt{{F}_{adh}}+\sqrt{F+{F}_{adh}})}^{2}]}^{1/3}$$

and where K:

5 $$K=\frac{4E}{3(1-{v}^{2})}$$

where E is the Young modulus of the sample, and ν the Poisson’s ratio of the sample. According to recent investigations^43^, a value of 0.4 for ν was used.

Thus, in the JKR model:

6 $$z={z}_{0}+d+\frac{{r}_{c}^{2}}{R}-\frac{4}{3}\sqrt{\frac{{r}_{c}{F}_{adh}}{RK}}$$

By using Eq. 6 to fit force curves in the cantilever deflection vs sample displacement representation it is possible to find the not only the contact point, z_0_, but also the Young modulus of the sample, E. Then, sample vertical position was converted to real probe-sample distance by adding the corresponding cantilever deflection:

7 $${d}_{ts}=(z-{z}_{0})+d$$

and subsequently to forces by using Eq. 1.

In accordance with previous studies^24, 25^, the bridging forces observed while approaching probe and sample were fitted with an exponentially decaying function:

8 $${F}_{bridging}({d}_{ts})=-{F}_{b0}\cdot \exp (-\frac{{d}_{ts}}{{\lambda }_{b}})$$

The adhesion observed when withdrawing probe and sample was characterized in terms of the adhesion energy per unit area. For this, first the work of adhesion, W_adh_, was obtained by computing the area under the baseline in the force curves. Then, W_adh_ was scaled by the maximum sample contact area in order to take into account differences in probe size and sample elasticity. Specifically, the contact radius at the maximum applied load, r_c_, was estimated from the fit of the corresponding approach force curve to the JKR model. Then the maximum sample contact area for each curve was calculated as:

9 $${A}_{{\rm{\max }}}=\pi \cdot {r}_{c}^{2}({F}_{{\rm{\max }}})$$

and the adhesion energy per unit area, w_adh_, was estimated as:

10 $${w}_{adh}=\frac{{W}_{adh}}{{A}_{{\rm{\max }}}}$$

Thus, for each force measurement we could estimate the Young modulus of the sample, the strength and characteristic length of the bridging interaction, F_b0_ and λ_b_, and the adhesion energy per unit area, w_adh_. As commented in the previous section, because of ageing effects and the long acquisition time needed for each measurement, only a small number of force curves could be obtained on each sample. As a consequence, the set of these quantities obtained from the analysis of the force measurement on a single sample could not be properly described by a normal distribution (with the corresponding mean and standard deviation values). Thus, median values and boxplot representations (where the central mark indicates the median, the bottom and top edges of the box indicate the 25^th^ and 75^th^ percentiles, respectively, and the whiskers extend to the most extreme data points) were used to present the distribution of the analyzed quantities. Finally, for analyzing the significance of the variations induced in these quantities by changes in the ambient pH, p-values from two sample t-tests were used.

### Data availability

The authors declare that the data supporting the findings of this study are available within the paper and its supplementary information files.

## Electronic supplementary material

Supplementary Information
