# Supplementary material for: Ex-Vivo Force Spectroscopy of Intestinal Mucosa Reveals the Mechanical Properties of Mucus Blankets
Source: Sci Rep. 2017 Aug 4;7:7270. doi: 10.1038/s41598-017-07552-7 (PMC5544714; doi:10.1038/s41598-017-07552-7)
Supplement: Supplementary file 1 — Supplementary Information [file 41598_2017_7552_MOESM1_ESM.pdf]

Ex-Vivo Force Spectroscopy of Intestinal Mucosa Reveals the Mechanical Properties of Mucus Blankets

- Supplementary Information -

Javier Sotres[1,2], Skaidre Jankovskaja[1,2], Kristin Wannerberger[3] and Thomas Arnebrant[1,2]

[1] Biomedical Science, Faculty of Health and Society, Malmö University, 20506 Malmö, Sweden.

[2] Biofilms-Research Center for Biointerfaces, Malmö University, 20506 Malmö, Sweden.

[3] Ferring International Center SA, CH-1162 St-Prex, Switzerland.

Supplementary Information, Section 1. Ileum samples extensively rinsed with deionized water

Ileum mucosa extensively rinsed with deionized water for ca. 1 min was also investigated. This procedure led to the complete removal of mucus blankets (Fig. S1a). A representative approach force measurement obtained on these samples in PBS pH 7.4 with a commercial AFM cantilever (OMCL-RC800PSA, Olympus, Japan, nominal force constant 0.05 $N \cdot m^{-1}$) is shown in Fig. S1b. No long-range bridging interactions were observed. No penetration of the samples took place either. Thus, force measurements were acquired by displacing the samples with a standard piezoelectric positioning system. Thus, we could obtain a significantly higher number of measurements than for mucus-covered tissues. The contact region of the curves was fitted with the JKR model (Eq. 6, Methods section, main text) for obtaining the Young modulus, E, of the samples. This provided a value for E of 17.1±2.2 kPa (distribution is plotted as an inset in Fig. S1b). This value is significantly lower than that obtained for ileum samples which preserved their mucus blankets (11-163 Pa, Results section, main text).

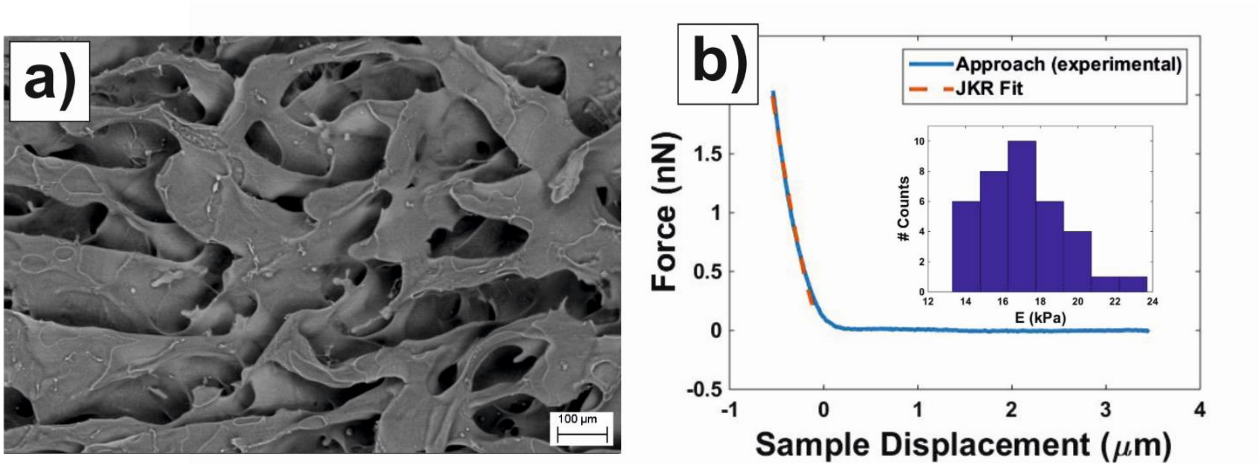

Figure S1. a) SEM image of a freeze-dried ileum mucosa sample which previously was extensively rinsed with deionized water. **b)** Representative force measurement (solid blue line) while approaching the mucosal surface in PBS pH 7.4 and corresponding JKR fir (red dashed line). The inset represents the distribution (n=36) of calculated Young moduli for a representative water-rinsed sample.

Supplementary Information, Section 2. Mucus removal by long exposure to physiological solution

It has been reported that most types of mucus are not chemically cross-linked gels, but physically entangled gels instead. This is, they can swell indefinitely. Our results indicate that this is the case for ileum mucus as well. As evidenced by SEM visualization, ileum samples that were incubated in PBS pH 7.4 for 4 h (in total 7 h after the sacrifice of the specimens) suffered a significant loss of their mucus blankets. The underlying villi could be resolved for one out of four samples treated in this way (the ratio increased along with incubation time)

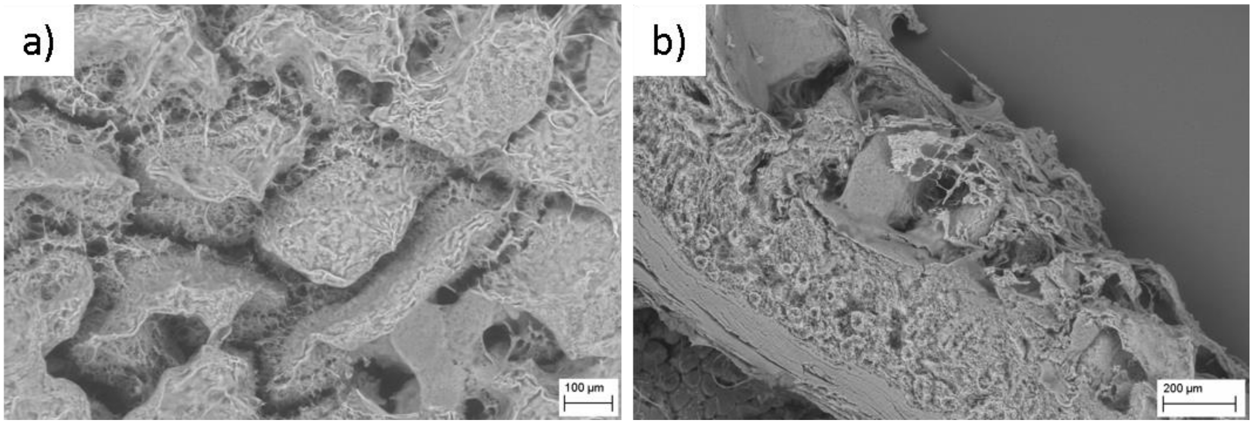

Figure S2. a) Top and **b)** section SEM images obtained in the variable pressure mode of freeze-dried ileum samples previously incubated for 4 h in PBS pH 7.4.

Supplementary Information, Section 3. Dependence of interaction parameters with acquisition time

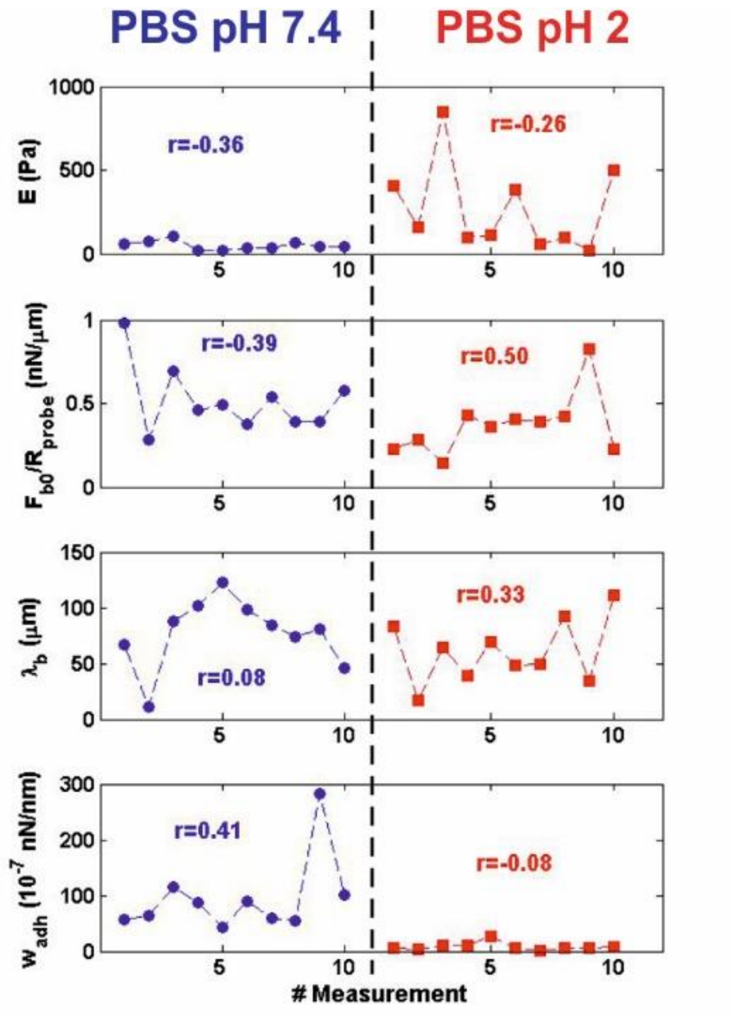

Figure S3. Young modulus (E), bridging force escalated amplitude, F_{b0}/R_{probe}, and characteristic length, λ_b, and adhesion energy per unit area, w_{adh}, calculated from a representative experiment on a single ileum sample versus the order of acquisition of the force measurements. Correlation coefficients between these quantities and the order of acquisition are also provided, and they indicate a non-significant influence of time in the experiments.
